# Implantable Cardiac Monitors: Evolution Through Disruption

**DOI:** 10.19102/icrm.2017.080903

**Published:** 2017-09-15

**Authors:** Chirag M. Sandesara, Rakesh Gopinathannair, Brian Olshansky

**Affiliations:** ^1^Department of Cardiology, Inova Heart and Vascular Institute, Falls Church, VA; ^2^Department of Cardiology, University of Louisville, Louisville, KY; ^3^Department of Cardiology, Mercy Hospital, Mason City, IA; ^4^University of Iowa Hospital and Clinics, Iowa City, IA

**Keywords:** Atrial fibrillation, implantable cardiac monitor, stroke, syncope

## Abstract

Syncope and stroke are commonly seen in clinical practice, and the diagnostic workup is often time-consuming and costly and may increase resource utilization in the health-care system. The use of implantable cardiac monitors (ICMs) in syncope evaluation has been well studied, but their use in cryptogenic stroke evaluation and anticoagulation management in patients with atrial fibrillation (AF) is still emerging. The standard workup of the syncope patient or those at risk for a possible cardioembolic stroke includes the utilization of external cardiac monitors; however, these devices cannot provide long-term arrhythmia assessment, whereas ICMs can now last up to three years, increasing the possibility of arriving at a diagnosis. Recent studies have shown that ICM use may shorten the time to diagnosis associated with AF, which may affect the prescribed treatment plan, thereby reducing the risks of further stroke. Long term and on a larger scale, this could potentially reduce overall health-care costs, but more studies are needed to confirm whether ICMs can positively decrease such costs and improve patient care. Still, these devices have become smaller and more reliable; additionally, they are now equipped with enhanced diagnostic capabilities, reducing the likelihood of physicians being confronted with an overwhelming amount of data, and supplying them with actionable items to improve patient care. With this growth, ICMs have in effect become a disruptive technology, as their applications in clinical practice continue to grow. Additional studies are warranted to investigate the safety and efficacy of their potential uses.

## Introduction

Patients who present with palpitations, syncope, or stroke may require cardiac monitoring for diagnostic assessment and therapeutic intervention when an arrhythmia is suspected to be the cause. However, the standard 12-lead electrocardiogram (ECG) provides only seconds of arrhythmia monitoring, which is often insufficient. Therefore, further arrhythmia monitoring is essential: while 24- to 72-hour ambulatory (ie, Holter) monitoring has potential value for those with frequent symptoms, patients with transient and intermittent symptoms require longer-term cardiac monitoring. External event recorders can assess asymptomatic and symptomatic events, quantitate arrhythmia duration, and allow for symptom/arrhythmia correlation, but the use of these devices is limited in many situations. Longer-term monitoring may be needed for transient and rare arrhythmia events that lead to troublesome problems, including undiagnosed syncope.

The implantable cardiac monitor (ICM) is a small device implanted subcutaneously into the left side of the chest, offering three to four years of battery life. Data are transmitted remotely, even on a daily basis, depending on arrhythmia capture. ICMs are most useful with infrequent, unexplained syncope, or if syncope is suspected to be of arrhythmic origin following an un-revealing initial workup.

ICMs have the advantage of long-term arrhythmia monitoring while also allowing for patients to self-capture and record symptomatic events. They may also be utilized to evaluate patients who have experienced an episode of stroke, in whom the cause of such is not otherwise identified, and atrial arrhythmias may be responsible. The ICM has technologically evolved over the past two decades based on the observed reduction in its size, ease of implant, and remote monitoring and data collection capabilities. As such, the utility of ICMs in the future may be disruptive and lead to broader applications such as QT monitoring following antiarrhythmic drug initiation and anticoagulation management in patients with intermittent atrial fibrillation (AF) who are at risk for stroke. However, these and other applications require vigorous clinical trials to better understand their potential in patient care. This review considers the role of ICMs in the clinical management of patients with syncope, stroke, and AF.

## Non-invasive cardiac monitoring for arrhythmia assessment

A carefully performed history and physical examination can usually identify the cause of syncope. However, despite the completion of an exhaustive investigation, a diagnosis is not always reached.^1^ Arrhythmias are a common cardiac cause for syncope. Recognizing and treating arrhythmias as the cause of syncope has clear value.^2,3^ Similarly, patients with suspected atrial or ventricular arrhythmias based on symptoms might be evaluated with event monitoring to determine arrhythmia type, duration, initiation, and termination. Clinical correlation with patient symptoms to the arrhythmia is critical for making an accurate diagnosis.

External loop recorders have been studied in the diagnostic evaluation of patients with a suspected arrhythmia that might lead to symptoms including palpitations, dizziness, or syncope. There are conflicting results regarding the ability to correlate a symptom to an arrhythmia, with efficacy ranging between 10% and 50%. External loop recorders are not designed for long-term monitoring, nor were they developed to store multiple recordings and, until recently, they have been cumbersome to wear.^4^ This technology is best-suited for patients with frequent, recurrent events occurring over days to weeks. Such standard monitoring devices are necessarily bulky to record a two- to three-lead electrocardiogram.

Wireless patch monitors are increasingly commonplace. The Zio^®^ Patch (iRhythm Technologies, Inc., San Francisco, CA, USA) is a United States Food and Drug Administration-approved adhesive, water-resistant, single-lead electrocardiographic sensor applied to the chest for 24-hour monitoring over two weeks. Its main limitations are its short battery life, which reduces long-term diagnostic possibilities, and the inherent difficulties in wearing it for an extended period of time. Furthermore, it is not P-wave centric and can demonstrate diagnostic problems for this reason.^5^

The SEEQ™ Mobile Cardiac Telemetry system (Medtronic, Inc., Minneapolis, MN, USA) involves a patch that is placed on the chest, which is replaced weekly with a new patch. A data collection center independently reviews alerts and notifies the clinician.^8^ In a retrospective study of 732 patients who underwent SEEQ™ (Medtronic, Inc.) monitoring, the time to detection of a clinically relevant arrhythmia (for example, bradycardia, pauses, second- and third-degree heart block, supraventricular tachycardia including sinus tachycardia, and AF), premature atrial and ventricular beats, and non-sustained or sustained ventricular tachycardia was 5.8 days.^9^ The Carnation Ambulatory Monitor™ (Bardy Diagnostics, Charlotte, NC, USA) is also available. It can record for up to a week, with high-quality signals that emphasize P-wave size and clarity. AliveCor Inc.’s (Mountain View, CA, USA) Kardia Mobile system involves a patient-centric, single-lead electrocardiogram performed via a smart-phone or tablet. It has unlimited storage, uses artificial intelligence to diagnose arrhythmias, and has similar efficacy to that of standard monitoring.^5^

These contemporary monitors have a growing role in cardiac event monitoring and are considered part of standard clinical practice by many as they increase patient compliance and provide real-time feedback on rhythm surveillance. However, their main limitation is their inability to acquire continuous data for months or years. They may also be costly for long-term successive use. Additionally, in the case of patient-centered technology such as the Kardia Mobile system (AliveCor Inc.), the risk for capturing false-positive events needs to be assessed. Thus, though looping event monitor evolution continues, with regards to long-term use, ICMs may provide improved arrhythmia surveillance for specific clinical situations and may be superior to external monitoring.

## ICMs

ICMs are single-channel electrocardiographic monitoring devices that allow for extended monitoring and provide a continuous long-term option for arrhythmia detection. The first such device made available, in 1998, was the Reveal™ ICM (Medtronic, Inc.), measuring 8 cc in size. ICMs have undergone several iterations via three separate manufacturers since then. The Confirm™ ICM (Abbott Laboratories, Chicago, IL, USA) is a 6.5-cc device that can store up to 18 months of AF data and provides episode duration and time/date stamps for recorded events. Similar to the Reveal™ ICM (Medtronic, Inc.), the Confirm™ ICM (Abbott Laboratories) is implanted into the left chest, lateral to the sternum, and offers a three-year battery life. Both devices are magnetic resonance imaging (MRI)-conditional, have remote-monitoring capabilities, and can store both auto- and patient-activated events. The BioMonitor 2 (Biotronik, Berlin, Germany) is a 5-cc device, implanted in the left chest; its battery longevity is four years. It provides space for more than 60 minutes of stored recordings for review, while sensing R-waves up to 1.7 mV. The limitations of these ICMs are the potential overdetection of atrial arrhythmias and underdetection of potential life-threatening arrhythmias. Myopotential oversensing may consume a significant amount of storage space.

**Figure 1:**
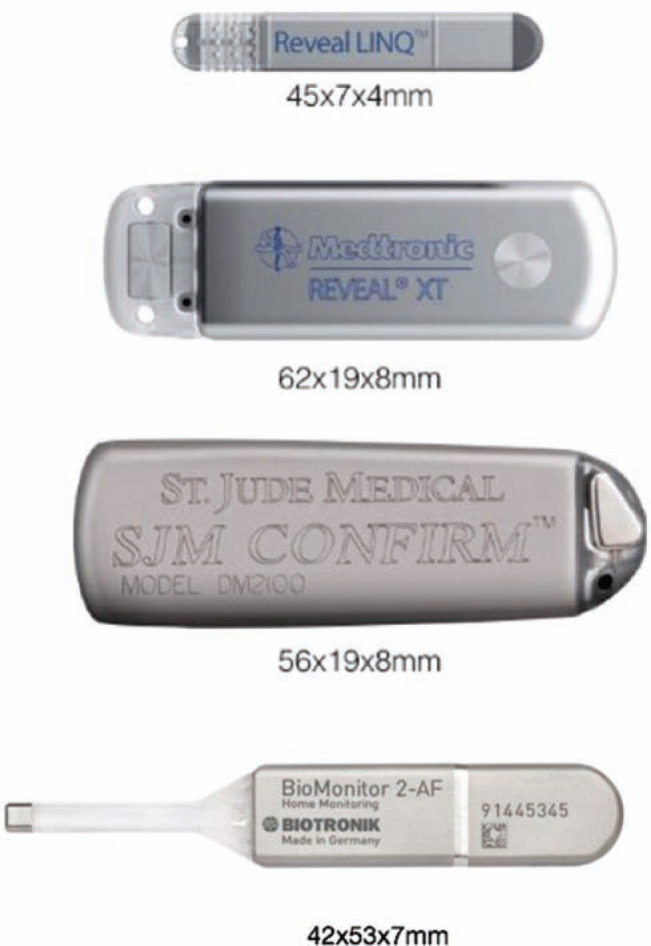
The Reveal LINQ™ ICM (Medtronic, Inc., Minneapolis, MN, USA) is much smaller than its predecessor, the Reveal XT™ (Medtronic, Inc., Minneapolis, MN, USA), the Confirm™ (Abbott Laboratories, Chicago, IL, USA) (third), and the BioMonitor 2 (Biotronik, Berlin, Germany) (bottom).

**Figure 2:**
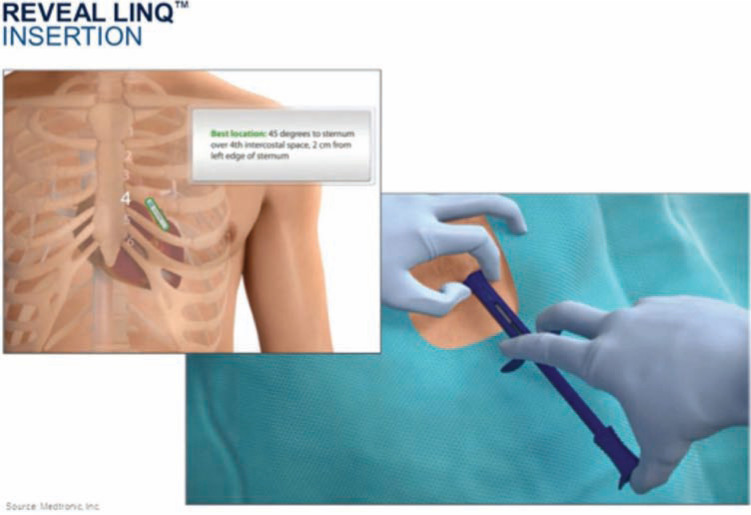
The Reveal LINQ™ (Medtronic, Inc., Minneapolis, MN, USA) device may be implanted within minutes. During the procedure, the skin is prepped/draped in a sterile fashion. A scalpel tool makes an incision at the fourth intercostal space, and the ICM device is inserted into the subcutaneous space. The incision may be sealed with skin glue, staples, or suture material.

**Figure 3:**
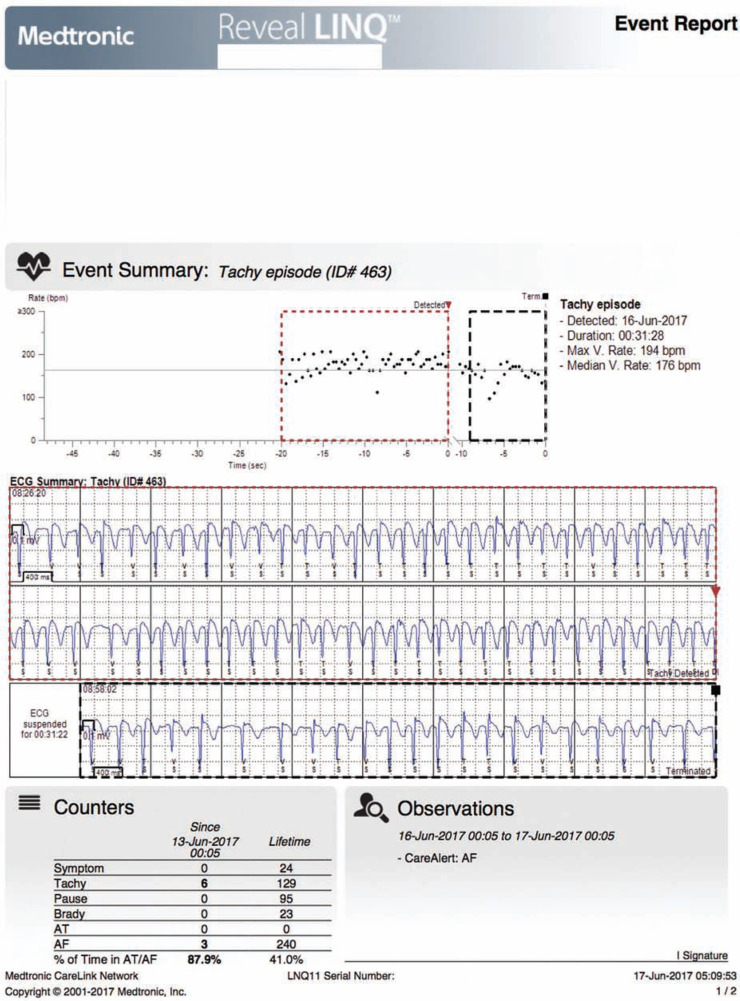

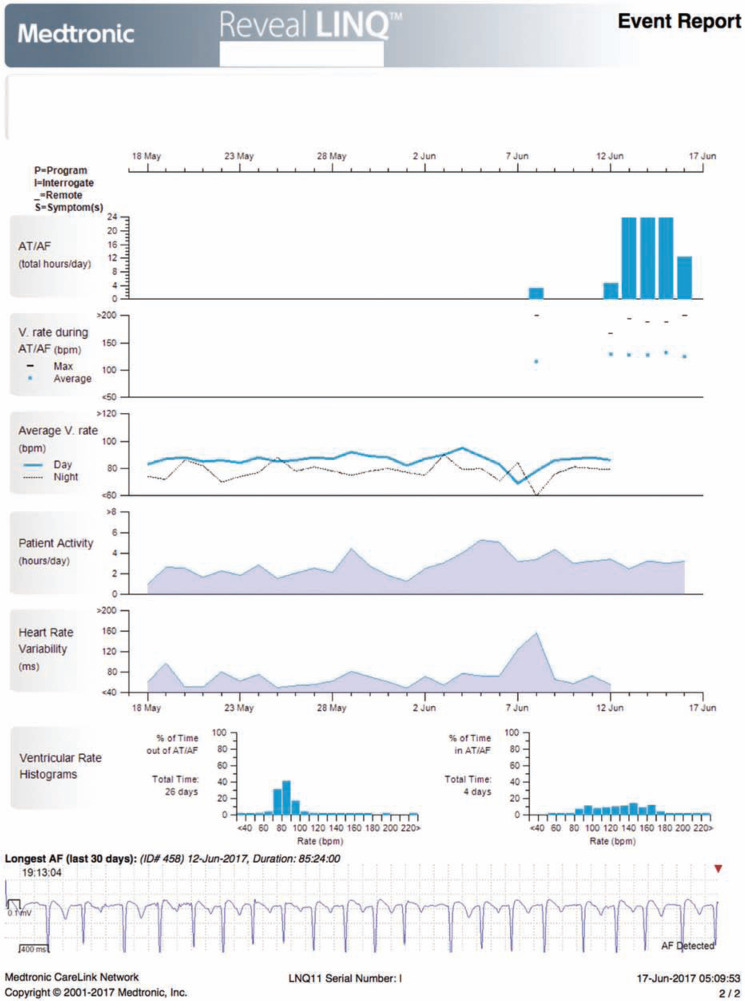
**A:** This tracing demonstrates an AF event. It shows the time of onset, duration, and patient heart rate. The percentages of time in AF, pause, and tachycardia/bradycardia events are also noted. **B:** The compass trend shows the previous AF events, along with heart rate changes and histograms.

**Figure 4:**
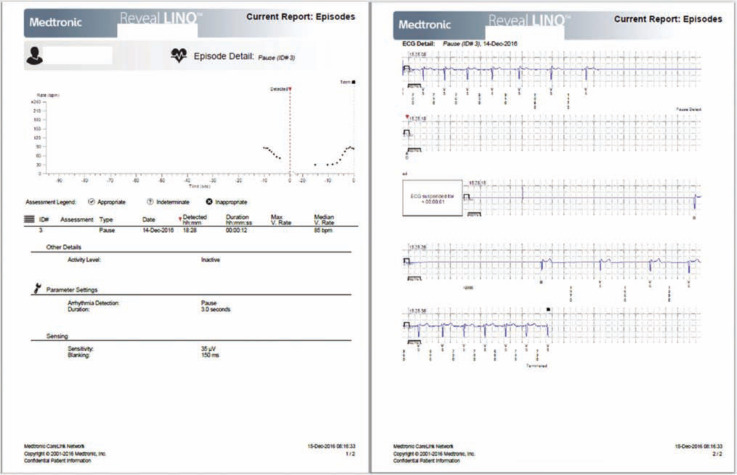
This event is an autocaptured pause lasting for 12 s. In this example, data collected 10 s prior to the pause are available for the clinician to review, along with those of 40 s thereafter.

The latest Reveal™ device is very small (Reveal LINQ™; Medtronic, Inc.), measuring ∼ 1 cc, or one-third the size of an AAA battery. This ICM is MRI-conditional at 1.5 and 3 Tesla, and provides up to three years of battery life **(Figure 1)**. Device insertion is simple, and only takes minutes to complete: during the procedure, the Reveal LINQ™ (Medtronic, Inc.) monitor is inserted through a tiny incision by hand injection into the subcutaneous tissue using a proprietary insertion tool **(Figure 2)**. Fifty-nine minutes of combined patient-activated and auto-activated events may be stored in the Reveal LINQ™ (Medtronic, Inc.), including AF, bradycardia, and pause events **(Figures 3 and 4)**. The Reveal LINQ™ (Medtronic, Inc.) also has enhanced filtering capabilities to reduce undersensing of pauses or bradycardia events, and a self-learning algorithm so that sinus arrhythmia is not labeled as a true AF event.

The Reveal LINQ™ (Medtronic, Inc., Minneapolis, MN, USA) device’s AF detection algorithm is based on R-R intervals and P-waves. It evaluates patterns in a Lorenz plot of R-R interval differences and also incorporates a P-wave “evidence score” by averaging a 600-ms baseline ECG window before R-waves for four-consecutive-beats meeting rate and irregularity criteria for AF. It then computes an AF “evidence score” every two minutes to determine if AF is present. The Reveal LINQ™ (Medtronic, Inc.) device’s algorithm was clinically studied in 202 patients, and it correctly identified 97.8% of the total AF duration and 99.3% of the total sinus of non-AF rhythm duration compared with Holter monitoring.^6^

A 30-day compass trend highlights pertinent data such as AF incidence, average heart rate, patient activity, and heart rate variability. Transmissions are sent via mobile phone towers and do not require a wired telephone line. It may act as an endless loop recorder and can automatically store bradycardia, tachycardia, and/or pause events based on prespecified patient-specific criteria. The patient may also self-record events for time and date correlation.

Microchip-sized injectable devices have been developed for AF evaluation but are not yet available for clinical use.^7^

### Indications for ICMs

The 2017 American College of Cardiology/American Heart Association/Heart Rhythm Society syncope guidelines indicate that ICMs may be considered for recurrent, infrequent, unexplained syncope or for use in patients with suspected arrhythmia-related causes of syncope following an initial non-diagnostic workup. These guidelines are applicable to patients with and without structural heart disease (Class IIa, LOE B-R).^8^

Insurance companies, however, allow for additional ICMs uses including cases of presyncope, questionable seizures, palpitations, and/or dizziness, particularly if the patients have structural heart disease or ECG abnormalities or if non-invasive event monitoring failed to make a diagnosis. They are especially useful in patients with infrequent or unpredictable symptoms.^9^

### ICMs for syncope management

The standard diagnostic evaluation methods for the syncope patient (external monitoring, imaging, echocar-diogram, stress testing, and tilt-table testing) can be time consuming and costly, as well as unproductive. ICMs, however, are also capable of establishing a cause of syncope, as has been shown in key clinical trials. The initial trial evaluating ICM efficacy in the clinical management of syncope was completed in 1995.^10^ This landmark study evaluated 16 patients with syncope in whom the baseline workup including a tilt-table test and an electrophysiology study was non-diagnostic. A syncopal event occurred in 15 (94%) patients over a period of four months; an arrhythmia was diagnosed by the ICM in 60% of patients.

The ISSUE trial examined 111 patients with unexplained syncope who were implanted with an ICM and followed for 3 to 15 months. A diagnosis of syncope was made in 29% of individuals in the ICM group versus 28% of individuals in the head-up tilt-table group. The most common cause of recurrent syncope in both groups was a prolonged asystole event that lasted 15 s on average.^11^

The ISSUE-2 trial was a non-randomized study of 392 patients with purported neurally mediated syncope events who underwent ICM implantation. Individuals with structural heart disease, ECG changes, and/or orthostatic hypotension were excluded. At one year post-implantation, data indicated syncope had occurred in 33% of patients. Enrollees with an ICM who had a recurrence of syncope due to arrhythmia went on to be implanted with a pacemaker or cardioverter-defibrillator, undergo ablation, or receive drug therapy as needed. Interestingly, the one-year syncope recurrence rate was markedly lower in the ICM-based therapy arm than in the standard care arm (10% versus 40%).^12^ Thus, ICMs appeared to guide effective therapy.

Another study examined 60 patients with syncope of unknown etiology who were treated with standard care (external event monitoring, tilt-table testing, and/or electrophysiology studies), versus an ICM.^13^ In this study, 52% of those in the ICM arm had a diagnosis (profound bradycardia, tachycardia, or pauses were noted at the time of syncope, based on the ICM recordings), compared to only 20% in the standard care arm. Other trials have shown ICMs to be superior to an otherwise-standard evaluation.^14,15^ These data indicate that prolonged monitoring was necessary to capture a diagnosis in syncope patients.

ICMs may also be beneficial in ruling out a cause of symptoms that may or may not be cardiac in nature. In a single-center retrospective evaluation, 53 (62%) patients had recurrent symptoms following ICM placement, with a mean time to recurrence of 12 ± 17 weeks. Of these, an arrhythmic diagnosis was established in 12 (145) patients. Forty-one (48%) did not have any arrhythmia during their symptoms.^16^

The FRESH study further supported the contention that ICMs have a critical role in syncope evaluation. In this prospective, open-label, multicenter study, low-risk syncope patients who underwent conventional evaluation for the condition were compared with those who underwent ICM implantation. A definite cause of syncope was more commonly found in the ICM group than in the conventional group (46% versus 5%; p < 0.001). Furthermore, less cardiac testing was performed in the ICM group, though there was no difference in reported quality of life between the two treatment arms.^17^

Remote ICM monitoring may affect patient treatments and outcomes. A retrospective observational study of 109 patients with ICMs compared the use of a strict in-home remote monitoring regimen to in-office visits completed once every three months. The mean times from implantation to diagnosis were 260 and 56 days for the in-office follow-up and remote monitoring arms, respectively (p < 0.01). This led to targeted treatment in the ICM group with remote monitoring being completed an average of 187 days earlier than in the in-office visit group, with no secondary complications.^18^

Further studies are needed to clarify how to best utilize ICMs in syncope patients and to determine methods to increase cost-effectiveness. A team approach incorporating someone who could remotely monitor patients and assess for alerts while providing both the clinician and patients with feedback might improve outcomes; however, further study is needed to assess the feasibility of this concept and to determine its direct impact on patient care. While syncope evaluation was the initial application of the ICM, it also has emerging roles in monitoring symptoms that signal the potential for an arrhythmia (such as palpitations), stroke patient assessment, and long-term AF management.

### ICMs in stroke assessment and prevention

The cause of a stroke can be obscure. ICMs have a potential role in helping clinicians understand the etiologies of certain stroke types and how they may affect treatment. Most strokes are ischemic in origin and occur largely due to atherosclerosis, small vessel disease, and/ or cardiac thromboembolism. Some strokes, however, have no definitive etiology, and are instead diagnosed as “cryptogenic,” based on the exclusion of all other known causes. Notably, 20% to 40% of strokes are cryptogenic, and may have one of several causes including AF, atherosclerosis, paradoxical embolism via an atrial septal defect, and/or *in situ* thrombosis due to a hypercoagulable state.^19–21^ One caveat is that a revealing condition may be present (eg, carotid plaque), but the cause may be due to some other mechanism (eg, a thromboembolic event due to AF).

Stroke risk is known to increase five-fold in patients with AF,^22^ and the risk of recurrent stroke is likewise increased if AF is present. However, AF may or may not necessarily have caused the stroke; instead, it could be due to other comorbidities such as hypertension, diabetes, or valvular heart disease, which may be present in the background of AF. AF may also be a signal indicating a sicker patient population that is characterized by having other stroke-relevant comorbidities. In light of this, the ability to potentially prevent stroke with oral anticoagulation is often missed.^23^ Certainly, anticoagulation therapy can be considered for all patients who have experienced an episode of cryptogenic stroke; however, this approach has an unfavorable risk/benefit profile due to the increased possibility of unnecessary bleeding complications.

Clinical trials investigating AF quantity and duration leading to a stroke have been completed. These trials employed the use of continuous arrhythmia monitoring via cardiac implantable electronic devices (CIEDs). The MOST study demonstrated that high-rate atrial events lasting more than five minutes that were characterized as AF via a pacemaker were associated with a 5.9-fold increased risk for clinically significant AF in the future, and carried a 2.8-times increased risk for the composite endpoint of stroke and death.^24^ The ASSERT trial studied an association between AF and stroke in 2,580 pacemaker/defibrillator patients who were over age 65 with no prior AF diagnosis. AF (defined as an atrial rate >190 bpm for more than six minutes) was found in 10.1% of patients over three months of follow-up. AF was also associated with a 2.5-fold increased risk of ischemic or embolic stroke in these patients.^25^ These studies may be extrapolated to ICMs, as they too may assist in AF detection in patients at risk for stroke.

A Veterans Administration health-care system study of 9,850 patients with a CIED evaluated the relationship between AF and stroke risk. AF duration in this study was 5.5 hours. Stroke risk was highest in the five days immediately following an AF event and decreased dramatically thereafter.^26^ Monitoring for atrial arrhythmias then may be pertinent in stroke prevention, but the impact of this on stroke recurrence and the risks for bleeding complications from anticoagulation in this population need to be considered and evaluated. It is important to recognize that not all studies have shown a temporal association between AF and stroke or that AF events are clearly associated with stroke.^27,28^

External cardiac monitors have been considered in the evaluation of cryptogenic stroke, but the obvious major limitation is the short duration of monitoring. Until ICM use was studied for this purpose, there was a gap in our ability to evaluate long-term, continuous data in patients after stroke, in whom a cardiac source may be the likely culprit. Previous studies have demonstrated an increase in AF detection with long-term monitoring in patients suspected of having an atrial arrhythmia as a cause of stroke.

The EMBRACE trial enrolled 572 patients who had experienced an episode of ischemic stroke of unknown cause in the previous six months but who had not been diagnosed with AF.^29^ These patients were randomized to a 30-day event monitor versus the standard workup and use of an additional 24-hour monitor. AF was detected in 16.1% in the event monitor group versus 3.2% in the standard work-up (control) group (95% confidence interval (CI), 8.0 to 17.6; p < 0.001). Oral anticoagulation therapy was prescribed to more patients in the monitoring group than in the control group (18.6% versus 11.1%, p < 0.01). There are limitations to this trial that affect the broad applications of anticoagulation for documented AF (≥30 s) after a stroke. The investigators concluded that causes other than AF might have instigated the stroke (not all patients had an extensive workup for non-AF causes of stroke). This study did not evaluate whether AF detection followed by anticoagulation affected stroke recurrence or increased bleeding risk.

The CRYSTAL-AF trial demonstrated a strong relationship between AF, duration of AF monitoring, and cryptogenic stroke.^30^ This randomized, controlled study of 441 patients was conducted to investigate if long-term ICM recording was more effective than conventional follow-up for detecting AF in patients with cryptogenic stroke. The primary endpoint was time to first detection of AF (> 30 s) within six months after stroke.

The incidence of AF was 8.9% in the ICM arm versus 1.4% in the control arm (hazard ratio (HR): 6.4, 95% CI, 1.9 to 21.7; p < 0.001). At 12 months, AF was diagnosed in 12.5% in the ICM group versus 2.0% in the control group (HR: 7.3, 95% CI, 2.6 to 20.8; p < 0.001). The median time to AF detection was 41 days in the ICM group and 32 days in the control group. In the ICM group, 10.1% of patients received an anticoagulant (at their clinician’s discretion), versus 4.6% of patients in the control group at six months (p = 0.04), and 14.7% versus 6.0%, respectively, at 12 months (p = 0.007). The rate of AF detection at 36 months was 30% in the ICM group versus 3% in the control group. There was less AF noted at 36 months versus 12 months in the control arm because only a few patients were followed for the full study duration. While this study showed that the presence of an ICM affected anticoagulation therapy use in patients in whom AF was detected, it did not evaluate the impact of anticoagulation on stroke recurrence or bleeding complications. For the EMBRACE and CRYSTAL-AF trials, outcomes associated with anticoagulation use based on events detected by an ICM remain unclear. These issues should lead to pause and speculation regarding the broad-based use of ICMs in patients with cryptogenic stroke until further trials are performed to address these important decision-driving clinical questions.

### ICMs for AF management following ablation

Limited data exist to guide the management of post-AF ablation patients regarding antiarrhythmic and anticoagulation medication use. ICMs may improve long-term AF detection and thus facilitate diagnostic and therapeutic decision making. This point-of-care approach would require intensive device monitoring and rigorous patient and physician communication to reduce the risk of AF recurrence or stroke if decisions regarding drug discontinuation are being made based on ICM data.

Zuern et al. studied 65 patients with CHADS2 scores of 1 to 3 who had undergone AF ablation and utilized an ICM over a follow-up of 32 ± 12 months. Of this population, 63% had AF for less than one hour per day and were able to remain safe without oral anticoagulation use, but 32% had to restart anticoagulants due to having an AF burden that exceeded one hour per day. There were no strokes or transient ischemic attacks with ICM use.^31^

Yang et al. studied 32 patients who underwent AF ablation, had an ICM implanted, and were followed for 24.7 ± 12.5 months. The ICMs recorded arrhythmic events in 18 (56.3%) patients including 12 with atrial arrhythmias (five recurred at nine, 12, 16, 17, and 32 months after ablation), two patients with ventricular tachycardia, and four with bradycardia.^32^

Kapa and colleagues assessed 44 patients undergoing AF ablation who received ICMs and cardiac monitors placed at 30 days, five months, and 11 months postablation. In the first six months, AF recurred in 18 patients (external cardiac monitoring and ICMs noted this in seven and 18 patients, respectively; p = 0.002). Five (28%) patients in the cardiac monitoring group and five (25%) in the ICM group had AF recurrence during the latter six months. AF was falsely diagnosed frequently by the ICM (730 of 1,421 episodes, 51%).^33^ This trial demonstrates a major limitation of ICMs; however, the impact of false-positive AF events was not further investigated.

ICMs may have a role in AF evaluation postablation. However, additional studies are needed to better define their role in such, and to determine if their use affects medical decision-making regarding drug therapy management.

## ICM evolution and simultaneous disruption

The role of ICMs continues to evolve with respect to AF detection. A recently published ASSERT substudy showed that tracking AF duration may relate to stroke incidence.^34^ In this report, 2,455 patients with CIEDs were followed for a mean of 2.5 years. The longest single episode of subclinical AF was from more than 6 minutes to 6 hours in 462 (18.8%) patients, from more than 6 to 24 hours in 169 (6.9%) patients, and more than 24 hours in 262 (10.7%) patients. The highest-risk subgroup included those patients in whom AF lasted more than 24 hours; these individuals had a significantly increased risk of subsequent stroke or systemic embolism (HR: 3.24, 95% CI, 1.51 to 6.95, p = 0.003).

Long-term (perhaps even lifelong) monitoring of patients with treated AF or those who have had an episode of cryptogenic stroke may increase the clinically important AF detection rate. However, the implications of long-term monitoring and its potential benefits are controversial. First and foremost, a longer-lasting ICM battery is needed for clinicians to be able to better understand the role of AF in cryptogenic stroke.

The need for longer-term monitoring is not clear, as the value of detecting an AF event years after a cryptogenic stroke is uncertain. The cost implications of an ICM in patients with a stroke are also enormous. Further studies are needed to better risk stratify this population and to determine who might most benefit from an ICM.

Some studies that truly disrupt and challenge our understanding of AF, anticoagulation use, and stroke have just been completed; others are still ongoing. The REVEAL-AF study presented at the Heart Rhythm Society’s 2017 Annual Scientific Sessions in Chicago aimed to assess if stroke risk predictors affect AF incidence in a non-AF population implanted with Reveal LINQ™ (Medtronic, Inc.) ICMs is one such study. This prospective, single-arm, multicenter trial included patients with a CHA_2_DS_2_-VASc score ≥ 3 or a CHADS2 score ≥ 2 and at least one of the following risk factors: coronary disease, renal disease (globular filtration rate: 30 to 60 ml/min), obstructive sleep apnea, and/or chronic obstructive pulmonary disease. AF was detected in 6.2% of patients at 30 days, which increased to 33.6% at 24 months. Interestingly, patient CHADS2 score did not affect AF incidence. The median time from device insertion to first detection was 123 days, and 56.3% of patients in whom AF was detected received oral anticoagulation at the clinician’s discretion.^35^

The ongoing ARTESIA study (NCT01938248) is evaluating the effect of oral anticoagulation on subclinical AF (asymptomatic AF lasting six minutes to 24 hours). The STROKE-AF trial is assessing the rate of AF in patients with a recent ischemic stroke via ICM over 12 months (NCT02700945). A recently completed but not yet published study identified and compared the rate and burden of AF (lasting more or less than 30 seconds) in patients with and without previous stroke, and assessed “how much” AF is necessary to increase the risk of stroke, how relevant the findings of AF are, and whether someone with AF should take anticoagulation therapy based on ICM data (NCT02843516). These and further studies are expected to shed light on the relationships among AF, stroke incidence, and anticoagulation management.

Risk prediction models such as the CHA_2_DS_2_-VASc scoring system and possibly the HAS-BLED score need rigorous evaluation and to be performed on an individual patient basis. For high stroke-risk patients, the potential for excessive anticoagulation leading to bleeding risk exists, especially in those with short runs or very infrequent episodes of AF. More studies are needed to better clarify the relationships among AF detection, duration, and stroke risk and to further assess whether anticoagulation improves outcomes in cryptogenic stroke patients when AF is detected or if AF is just one of several comorbidities associated with but not necessarily causal of stroke.

The evolution of ICMs heralds the advent of new technology that may increase patient accountability regarding event transmissions and truly disrupt how data are evaluated and transmitted by the patient. The first smartphone-compatible ICM (not currently available on the US market) could transform the way patients communicate data to physicians. The Confirm™ Rx ICM (Abbott Laboratories) will utilize Bluetooth wireless technology to allow smartphones to store and transmit arrhythmia data via an app. This 1.4-cc MRI-conditional device will eliminate the need for a handheld patient activator and bedside transmitter. This will also allow for alerts for patient-triggered symptoms to be immediately transmitted to the clinic. In addition, symptom keywords such as “faint” or “palpitations” may be transmitted with recordings to improve the clinical correlation of each event. Patient-centric app-based technologies like this will hopefully provide a platform for increased patient autonomy and accountability regarding their arrhythmia management. It remains to be seen whether this approach will improve outcomes in patients with syncope, AF, and/or other suspected arrhythmias.

## The future role of ICMs

The future role of the ICM as a diagnostic tool is highly favorable. It could be used for QTc measurements while titrating antiarrhythmic medication. ST segment changes could be assessed and could possibly alert the clinician prior to myocardial infarction. Non-invasive thoracic impedance changes could be incorporated into the ICM to better guide in-home heart failure management. Lastly, as the ICM could provide feedback to arrhythmia management via an app, future renditions of this technology may be a tool for patient-centered heart rate assessment, daily steps taken, metabolism measurement, blood sugar monitoring, and/or as a non-invasive blood pressure measurement device.

The technology could also provide important physiological data in a wide range of patients with various clinical conditions. The ICM could become more complex and provide a better diagnostic approach to the patient with syncope and associated symptoms. There is no reason why the monitors need to be limited to providing heart rate information.

In the meantime, however, there are still some limitations inherent in ICMs in terms of arrhythmia monitoring. These include their potential to record noise and their finite amount of storage.

Still, the ICM could become a disruptive technology once we fully understand its potential, though a price tag comes with every new advent in technology. Therefore, the question to consider is whether ICM data could be meaningful, actionable, and cost-effective. Existing data suggest an expanding role of ICM use in various clinical situations.

## Conclusions

ICMs have a growing role in evaluating patients with undiagnosed syncope, palpitations, and other suspected arrhythmic symptoms. Their use is evolving for the management of AF and assessing cryptogenic stroke. Otherwise, these conditions pose major diagnostic and therapeutic challenges. ICMs have been evaluated in these clinical contexts, and they may have a larger role to play in shortening the time to diagnosis and ultimately improving clinical outcomes. Novel functions for ICMs continue to emerge, and this movement will likely eventually disrupt our routine methods of evaluating and managing patients with suspected arrhythmias that presently cause diagnostic challenges.
